# The effects of diabetes and/or peripheral neuropathy in detecting short postural perturbations in mature adults

**DOI:** 10.1186/1743-0003-7-44

**Published:** 2010-09-13

**Authors:** George D Fulk, Charles J Robinson, Sumona Mondal, Christopher M Storey, Anne M Hollister

**Affiliations:** 1 Department of Physical Therapy, Clarkson University, Potsdam, NY, USA; 2 Center for Rehabilitation Engineering, Science and Technology, Clarkson University, Potsdam, NY, USA; 3 Department of Math and Computer Science; Clarkson University, Potsdam, NY, USA; 4 Research Service, VA Medical Center, Syracuse, NY, USA; 5 Department of Physical Med. & Rehab, Upstate Medical University, Syracuse, NY, USA; 6 Medical School, Louisiana State University Health Sciences Center, Shreveport, LA, USA; 7 Department of Orthopaedic Surgery, Louisiana State University Health Sciences Center, Shreveport, LA, USA

## Abstract

**Background:**

This study explored the effects of diabetes mellitus (DM) and peripheral neuropathy (PN) on the ability to detect near-threshold postural perturbations.

**Methods:**

83 subjects participated; 32 with type II DM (25 with PN and 7 without PN), 19 with PN without DM, and 32 without DM or PN. Peak acceleration thresholds for detecting anterior platform translations of 1 mm, 4 mm, and 16 mm displacements were determined. A 2(DM) × 2(PN) factorial MANCOVA with weight as a covariate was calculated to compare acceleration detection thresholds among subjects who had DM or did not and who had PN or did not.

**Results:**

There was a main effect for DM but not for PN. Post hoc analysis revealed that subjects with DM required higher accelerations to detect a 1 mm and 4 mm displacement.

**Conclusion:**

Our findings suggest that PN may not be the only cause of impaired balance in people with DM. Clinicians should be aware that diabetes itself might negatively impact the postural control system.

## Background

Complications associated with diabetes are linked to increased postural sway, slowing of peripheral sensory and motor pathways, abnormal neuromuscular response to postural disturbance, increased whole body reaction time, and abnormal gait patterns over irregular surfaces[1-3]. These complications may lead to impaired postural control and play a role in the increased risk of falling in this population[4].

Various authors have found that individuals with diabetes and peripheral neuropathy demonstrate impaired postural control in quiet standing compared to healthy control subjects. Boucher and colleagues[1] found that individuals with diabetes and peripheral neuropathy had greater postural sway in quiet standing and greater difficulty integrating sensory information for balance control than healthy control subjects. They also found that postural control was related to the severity of peripheral neuropathy. Lafond and colleagues[2]found that postural sway in elders with diabetes and peripheral neuropathy with eyes open was comparable to healthy elders with eyes closed. These studies focused on how peripheral neuropathy related to diabetes affected postural control.

Other authors have examined the impact of diabetes alone on postural control. In a group of young adults with insulin dependent diabetes mellitus (IDDM) both with and without peripheral neuropathy, Uccioli and colleagues[5] found significant differences in measures of static postural sway between subjects with IDDM with peripheral neuropathy and healthy controls. However, there was no difference in static postural control between subjects with IDDM without peripheral neuropathy and healthy controls. Incorporating somatosensory and motor evoked potentials this same group found that IDDM might affect both sensory and motor peripheral pathways, but only sensory pathways centrally[6].

Although peripheral neuropathy is commonly thought to be the cause of postural instability in people with diabetes, there is some evidence that diabetes *per se *may have a negative impact on postural control under more stressful conditions than quiet stance[7-10]. During a dynamic reaching task, Centomo and colleagues[9] found a significant difference in measures of postural control between middle-aged adults with diabetes without peripheral neuropathy and healthy control subjects. While standing with eyes closed and head back, Oppenheim and colleagues[8] found that individuals with diabetes without peripheral neuropathy had impaired postural control compared to healthy individuals. Recently, Allet and colleagues[7] found that people with diabetes without peripheral neuropathy demonstrate an abnormal gait pattern compared to healthy people. They also found that there was no difference in gait parameters between diabetics with and without peripheral neuropathy. Thus, peripheral neuropathy associated with diabetes may not be the only factor contributing to impaired postural control in people with diabetes.

Only 30% of people with diabetes have peripheral neuropathy[11,12]. This leaves seventy percent of people with diabetes who may also demonstrate abnormal postural control, but may not be identified by clinicians as having poor balance because they do not have peripheral neuropathy. Because of the growing evidence that diabetes itself may negatively impact balance and increase fall risk, further research exploring the impact of diabetes on postural control is necessary. Our lab examines quasi static posturography where we deliver via a sophisticated surface translational platform perturbations that are in the range of normal postural sway Root-Mean-Square (RMS) path length. In this way we can investigate the control mechanisms of the postural control system without overtly generating a fall initiating response. We have previously found that older individuals with diabetes have a significantly longer reaction time to threshold perturbations than individuals without diabetes to anterior translations[13]. Thus the purpose of this study was to explore separately and jointly the effects of diabetes and peripheral neuropathy on the ability of individuals to detect perithreshold anterior postural perturbations.

## Methods

This psychophysical research described here is a part of an extensive protocol in use in our lab to:

A. Psychophysically determine by iteration the acceleration values (i.e., the detection thresholds) at which fixed-length anterior horizontal platform translations of 1, 4 and 16 mm can be detected; response latencies to peri-threshold and super-threshold translations; thresholds and reaction times to foot-sole touch; and thresholds and reaction times to tone pulses[13].

B. Biomechanically measure changes in platform position and acceleration, and in the center-of-pressure of the subject as projected onto a force plate, head acceleration via a tri-axial accelerometer, and horizontal ground reaction force.

C. Neurophysiologically measure changes in lower limb gastroc/soleus and tibialis anterior EMGs brought about by perturbation.

This paper deals only with the psychophysical part of the protocol (A above), its methodology and results from adult subjects at or over 50 years of age.

### Subjects

Subjects were recruited through approved flyers posted in the Overton Brooks VA Hospital in Shreveport, Louisiana, and the surrounding communities. Approximately half of the subjects were patients at the VA hospital and the other half from the surrounding communities. All subjects provided informed consent and the institutional review boards at the Shreveport VAMC and Louisiana Tech University approved the study protocol. The subject's primary care physician made the diagnosis of type II diabetes mellitus and presence or absence of peripheral neuropathy was determined by nerve conduction velocity (NCV) testing (see below for details). Subjects were classified as diabetics with peripheral neuropathy (DPN), neurologically intact diabetics without peripheral neuropathy (DNI), non-diabetics with PN (PNNoD), and neurologically intact adults without DM (NInoD). The exact cause of PN in the subjects without diabetes with PN was not known.

All subjects underwent a visual, auditory, musculoskeletal, and cognitive screening to ensure that they had no undiagnosed condition that may have affected their balance. Subjects with respiratory dysfunction, cardiac condition, central nervous system disorder, musculoskeletal disorder, lower extremity amputation, severe arthritis, history of repeated falls, or currently taking medication to prevent dizziness were excluded.

### Instrument

Balance capability was measured using the Sliding Linear Investigative Platform for Assessing Lower Limb Stability (SLIP-FALLS), a horizontal translating force platform and data collection system[14]. The SLIP-FALLS platform was specifically designed and built to assess psychophysical thresholds to postural perturbations. This highly instrumented platform and its controller enable investigators to precisely control the platform displacement and acceleration. The use of a non-contact linear motor and air bearing slides essentially eliminates vibration during movement of the platform, thereby eliminating extraneous cues to the subject that the platform is being moved. Postural sway parameters (anterior-posterior and medial-lateral center of pressure) are calculated from the four load cells of the force-platform[15].

### Procedure

Using an adaptive 2 alternative forced choice (2AFC) protocol[16], the acceleration thresholds for detecting an anterior-posterior, horizontal translation of the platform at displacements of 1 mm, 4 mm, and 16 mm were determined in separate runs of up to 30 trials each. Peak platform acceleration was the variable iterated to threshold. During the first half of the move the platform was smoothly accelerated under precise control; and in the second half, it was smoothly decelerated, in both cases so that jerk is minimized. Peak acceleration was programmed to occur one-forth of the way into the move, zero acceleration at the middle of the move, and peak deceleration, three quarters into the move. These smoothed acceleration profile produced a much subtler move than one that immediately turns on and maintains a fixed peak acceleration at the start, and then suddenly reverses it to a fixed peak deceleration during the second half of the move, with concomitant high jerk at the beginning, middle and end of the perturbation[13,14].

While standing barefoot and blindfolded on the SLIP-FALLS a subject was presented with the commands "Ready", "One", "Two", "Decide" via headphones, through which masking white noise (70 dB SPL) was additionally presented. The time intervals for "Ready" and "Decide" were 4 and 3 s, respectively. For platform movements of 1 mm and 4 mm, the time interval was 4 s and or the 16 mm platform movement, the time interval was 6 s. During the interval "One" or "Two", the platform moved a fixed displacement (1 mm, 4 mm, or 16 mm) at a test acceleration. After the word "Decide", the subject was *required *(i.e., the choice was forced) to press a handheld button once or twice to signify in which interval he/she perceived the perturbation to have occurred. Platform movement was pseudo-randomly assigned to occur in either interval "One" or "Two", ensuring that an equal number of platform movements occurred in each interval.

A modified Parameter Estimation by Sequential Testing (PEST) algorithm[17,18] was used to determine the acceleration threshold for perception of movement at a given displacement (1 mm, 4 mm, and 16 mm). This algorithm changed the platform acceleration from one trial to the next as the acceleration was iterated towards detection threshold. The modified PEST methodology ensured that all perturbations were near, or rapidly, approaching within 30 trials in order to prevent fatigue[16,18,19]. This technique reduces the number of measurements needed to converge to threshold. The PEST target probability is set at a level of change rather than a percentage of "correct" responses. This protocol was designed so that an individual subject would accrue a correct detection percentage of 79% for test accelerations at threshold[16]. In psychophysical testing 75% is the generally accepted criteria for psychophysical detection[16].

**Figure 1:**
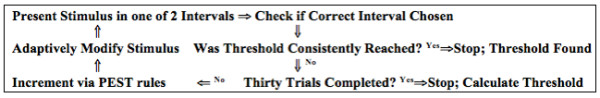
**Iterative Protocol for Estimation of the Detection Threshold via the 2-Alternative-Forced Choice and Parameter Estimation by Sequential Testing Procedures**.

The displacement order (1 mm, 4 mm, or 16 mm) was randomized. A 10 to 15 minute rest period was taken after each acceleration threshold was identified at a fixed displacement before moving on to the next displacement. For example, after the acceleration threshold was identified in at most 30 trials at the first test displacement (e.g., 1 mm), the subject rested for 10 to 15 minutes before beginning another 30 trials at a different displacement (e.g., 4 mm or 16 mm). Figure 1 provides an overview of the psychophysical 2AFC PEST protocol. Further details of and justification for the experimental 2AFC PEST psychophysical test paradigm that was developed for the SLIP-FALLS lab and used here can be found in Richerson et al[20].

Because the perturbations were very short and accelerations well below that employed by any other research or commercial perturbation platform tests[14], our subjects stood without external support (i.e., safety harness) during all testing. Because the PEST rules are such that a series of successive misses in one interval (or correspondingly false positives in the other), would lead to ever increasing acceleration levels, our protocol needed to limit the maximum peak acceleration that could be used under a given displacement. These peak (or rail) levels were originally set to well exceed any threshold found in our original young adult population. Rails were set at 200, 100, and 100 mm/s^2 ^for displacements of 1, 4, and 16 mm respectively. The modified PEST protocol would not allow acceleration values to exceed these levels, but if three correct decisions were made in a row, the algorithm would decrease the test acceleration in the next trial to a value below the maximum. The rail values were thus ceilings that could not be exceeded, but that could be visited briefly or for the remaining duration of the 30 trial runs. It became apparent early in these experiments that a subject's behavior was such that we sensed (but could not prove) in some individuals that threshold was very near or slightly above the rail values initially used. As such we modified the rail values to be set to 362, 256, and 181 mm/s^2 ^(i.e., 2^8.5^, 2^8^, and 2^7.5 ^mm/s^2^) for moves of 1 mm, 4 mm, and 16 mm for all subjects.

Previously our lab has found test-retest reliability for psychophysical detection of movement with individuals with and without diabetes using our 2AFC PEST protocol described above to be ICC_2,1 _to be 0.645 (P < 0.05)[21]. Intervals between testing ranged from the same day to two weeks.

In addition to acceleration threshold detection data gathered with the SLIPP-FALLS, the following data were also gathered for each subject: Berg Balance Scale (BBS) score, Semmes-Weinstein Monofilament (SWM) touch detection thresholds, and surface lower-limb nerve conduction velocities (NCV). The maximum score on the Berg is 56; a score below 40 indicates a fall risk. The Berg has been shown to have excellent interrater (ICC = 0.91) and test-retest (ICC = 0.92) reliability and concurrent validity for older individuals[22,23]. No reports could be found that examined the psychometric properties of the BBS in people with type II diabetes mellitus.

Sensory testing was performed with SWM on the plantar surface of the great toe, plantar surface of the metatarsal of the first and fifth toes, and the heel. Semmes Weinstein monofilaments have high reliability and validity for determining sensory impairment in people with diabetes[24,25]. A trained research assistant performed Berg Balance and SWM testing.

A trained clinical neurology technician performed surface lower-limb nerve conduction testing in the neurology clinical suite, and a neurologist supervised and interpreted the tests. Subjects were classified as having peripheral neuropathy based on normative data used by the neurology department at the Pittsburgh VAMC. Nerve conduction velocities (NCV) were measured for the tibial, peroneal, and sural nerves bilaterally, and the thresholds set as abnormal were at or below 41 m/s, 44 m/s, and 34 m/s respectively. Each NCV was normalized by its threshold value, and the overall NCV score *X *was set to the minimum of the normalized velocities. Subjects were classified as having peripheral neuropathy when *X *< = 0.98 and as being neurologically intact when *X *> = 1.02. If a subject's *X *score fell in the ± 2% gap (0.98 <*X *< 1.02) they were excluded from the data analysis. It was felt that including a gap would provide a more reliable classification in comparison with classifying every subject as either having peripheral neuropathy or being neurologically intact, even when the NCV value fell right on the boundary.

### Data Analysis

To determine if differences in subject characteristics were due to disease status (diabetes vs. no diabetes and peripheral neuropathy vs. no peripheral neuropathy) factorial ANOVAs were used to examine differences for age, weight, BMI, BBS scores, NCV testing and SWM testing. This analysis revealed a significant difference in weight between those with diabetes and without diabetes (see below). Due to this difference weight was used as a covariate in our subsequent analyses.

A 2(diabetes) × 2(peripheral neuropathy) between-subjects factorial MANCOVA was calculated to compare acceleration detection threshold at 1 mm, 4 mm and 16 mm displacements for subjects who had diabetes (DPN and DNI subjects) or did not (PNNoD and NINoD subjects) and who had peripheral neuropathy (DPN and PNNoD subjects) or were neurologically intact (DNI and NINoD subjects) with weight as a covariate. Since we have unequal sample sizes in the different groups, data from each group was tested for normality and equality of variance to establish group equivalences necessary for using a MANCOVA using a Generalized Linear Model approach, and these criteria were met (p > 0.05). Alpha was set at 0.05 for all analyses.

Multifactor ANOVA studies are conducted when we need to investigate the simultaneous effects of two or more factors on one or more output variables (i.e. response variables). In this case the two factors are diabetes and peripheral neuropathy. The response variables are the acceleration detection thresholds at the three different distances (1 mm, 4 mm, and 16 mm). This method is powerful, efficient and provides information not only of the main effects of the factors but also of their combined interactions. Since we have unequal sample sizes, to satisfy the orthogonality of the MANOVA decomposition, the general linear test approach was used in our experiment for two different factors (diabetes and peripheral neuropathy). Moreover, to reduce the variance in the error term we augmented the MANOVA model with the covariate of weight. These quantitative variables are related to our response variables (1 mm, 4 mm, and 16 mm). These analogies lead us to use multifactor analysis of variance with covariate to obtain the optimum analysis for our data set.

## Results

Eighty-three subjects between the ages of 50 and 77 were recruited for this study. Thirty-two were diagnosed with type II diabetes mellitus -- 25 of them had verified peripheral neuropathy (DPN) and seven were neurologically intact without peripheral neuropathy (DNI). Nineteen subjects were diagnosed with PN without diabetes (PNNoD) and 32 subjects were neurologically intact adults without diabetes (NINoD).

Since the purpose of this paper was to find correlates for detection threshold levels, subjects who could not reliably iterate to threshold values on these tests were eliminated from further analysis. There were 14 such subjects who were not able to identify an acceleration threshold prior to reaching the rail values at two or more displacements (seven DPN, five PNNoDM, and 2 NINoD). This resulted in a total of 69 subjects that were used in the data analysis (Table 1).

**Table 1:** Subject Characteristics

Group	N	Age Mean (std)
DPN	18	60.8 (6.6)

DNI	7	58.1 (7.2)

PNNoD	14	57.8 (6.3)

NINoD	30	58.4 (7.4)

The 2 × 2 factorial ANOVAs (with/without diabetes × with/without peripheral neuropathy) comparing subject characteristics found no significant difference in age, BMI, right foot sensation, and left sural nerve conduction velocity among groups (p > 0.05). A significant difference was found among groups (p < 0.05) for weight, BBS, all nerve conduction velocities (except left sural) and left foot sensation. Individuals with diabetes weighed significantly more, 207.6 (± 38.9) lbs, than those without diabetes, 179.4 (± 38.1) lbs (p < 0.05). Individuals with diabetes scored lower on the BBS, 55.7 (± 0.63), than subjects without diabetes, 56.0 (± 0.00) (p < 0.05). Scores for individuals with diabetes ranged from 54 to 56, while all the subjects without diabetes scored 56. Due to test-retest reliability of the BBS, this small difference in mean scores is not likely clinically meaningful.

Individuals with peripheral neuropathy demonstrated significantly slower nerve conduction velocities in both the right and left lower extremity in all three nerves tested except for the left sural nerve (Table 2). Individuals with diabetes required greater force to detect a sensory stimulus than individuals without diabetes at the left great toe, base of the left first metatarsal, base of the left fifth metatarsal, and left heel. Contrary to our expectations there were no significant differences in SWM testing results between individuals with PN and without PN (Table 3).

**Table 2:** Nerve Conduction Velocity Testing

Peripheral Nerve	Peripheral Neuropathy Group Mean (std) N = 32	Neurologically Intact Group Mean (std) N = 37	Diabetic Group Mean (std) N = 25	NonDiabetic Group Mean (std) N = 44
Left Peroneal (m/s)	41.69 (3.98)*	48.00 (2.95)	42.72 (4.04)	46.41 (4.52)

Left Tibial (m/s)	42.06 (3.76)*	46.92 (3.29)	42.84 (4.64)	45.70 (3.68)

Left Sural (m/s)	42.20 (5.74)	43.88 (4.19)	42.11 (6.14)	43.66 (4.24)

Right Peroneal (m/s)	41.97 (3.98)*	47.84 (2.84)	42.40 (4.68)**	46.66 (3.60)

Right Tibial (m/s)	41.50 (3.19)*	46.59 (4.29)	42.40 (4.15)	45.27 (4.52)

Right Sural (m/s)	41.08(4.01)*	44.22 (3.62)	42.00 (4.84)	43.26 (3.64)

* significant main effect for subjects with peripheral neuropathy versus those without peripheral neuropathy, p < 0.05; ** significant main effect for subjects with diabetes versus subjects without diabetes

**Table 3:** Semmes Weinstein Monofilament Sensory Testing

Testing Location	Peripheral Neuropathy Group Mean (std) N = 32	Neurologically Intact Group Mean (std) N = 37	Diabetic Group Mean (std) N = 25	Non Diabetic Group Mean (std) N = 44
Left Great Toe (log of force in grams)	3.85 (0.63)	3.56 (0.48)	3.95 (0.56)*	3.55 (0.53)

Base of Left 1^st ^Metatarsal (log of force in grams)	4.06 (0.56)	3.70 (0.62)	4.14 (0.70)*	3.71 (0.51)

Base of Left 5^th ^Metatarsal (log of force in grams)	4.26 (0.76)	3.85 (0.62)	4.41 (0.65)*	3.87 (0.70)

Left Heel (log of force in grams)	4.60 (0.75)	4.42 (0.54)	4.99 (0.58)*	4.24 (0.54)

Right Great Toe (log of force in grams)	3.73 (0.71)	3.64 (0.50)	3.87 (0.68)	3.57 (0.53)

Base of Right 1^st ^Metatarsal (log of force in grams)	3.97 (0.58)	3.67 (0.48)	3.97 (0.65)	3.72 (0.47)

Base of Right 5^th ^Metatarsal (log of force in grams)	4.22 (0.65)	3.86 (0.56)	4.29 (0.82)	3.91 (0.44)

Right Heel (log of force in grams)	4.65 (0.63)	4.41 (0.54)	4.71 (0.54)	4.44 (0.61)

* significant main effect for subjects with diabetes versus those without diabetes, p < 0.05

**Figure 2:**
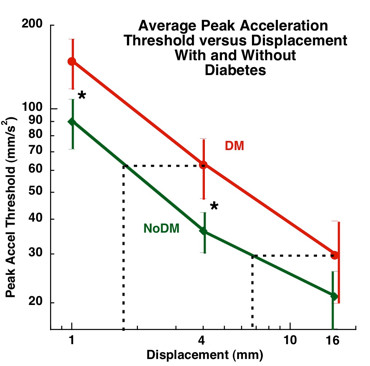
**Relationship Between Test Displacement and Peak Acceleration Threshold in Subjects with (N = 25, red line) and without Diabetes (N = 44, green line)**. The "*" indicates a significant group effect for subjects with diabetes versus those without diabetes at 1 mm and 4 mm displacements. The lines connecting the means illustrate significant differences in acceleration thresholds between displacements of 1, 4 and 16 mm. The horizontal dotted iso-acceleration lines demonstrate that individuals with diabetes would need approximately twice the perturbation length to detect a whole body movement at the same acceleration as individuals without diabetes.

**Figure 3:**
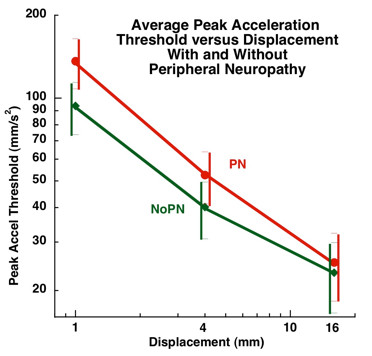
**Relationship Between Test Displacement and Peak Acceleration Threshold in Subjects with (N = 32, red line) and without Peripheral Neuropathy (N = 37, green line)**. There was no significant group effect for subjects with PN versus those without PN at any displacement.

For the acceleration detection threshold testing, the factorial MANCOVA analysis revealed a significant main effect for diabetes (Figure 2), but none for peripheral neuropathy (Figure 3). Subjects with diabetes required higher accelerations to detect a displacement than subjects without diabetes, p < 0.05. Tukey's post hoc analysis revealed that subjects with diabetes required higher accelerations to detect 1 mm and 4 mm displacements than subjects without diabetes. Subjects with diabetes required an acceleration of 148.2 mm/s^2 ^(95% CI: 118.0-178.4) to detect a 1 mm whole body displacement, while subjects without diabetes only required an acceleration of 89.8 mm/s^2 ^(95% CI: 71.4-108.2). Subjects with diabetes required an acceleration of 62.8 mm/s^2 ^(95% CI: 47.4-78.2) to detect a 4 mm whole body displacement, while subjects without diabetes only required an acceleration of 36.31 mm/s^2 ^(30.3-42.3). There was no significant group difference in acceleration detection threshold at a 16 mm displacement. Subjects with diabetes required a 29.7 mm/s^2 ^(95% CI: 19.9-39.4) acceleration to detect a 16 mm whole body displacement versus a 21.1 mm/s^2 ^(95% CI: 16.1-26.0) acceleration for subjects without diabetes.

There was no significant difference in acceleration threshold detection between subjects with peripheral neuropathy and those without peripheral neuropathy at any of the three whole body displacement distances, p < 0.05 (Figure 3). In order to detect a 1 mm whole body translation, subjects with peripheral neuropathy required an acceleration of 136.2 mm/s^2 ^(95% CI: 108.1-164.3) and subjects without peripheral neuropathy required an acceleration of 93.7 mm/s^2 ^(95% CI: 73.4-114.0). In order to detect a 4 mm whole body translation, subjects with peripheral neuropathy required an acceleration of 52.5 mm/s^2 ^(95% CI: 41.3-63.8), while subjects without peripheral neuropathy required an acceleration of 40.2 mm/s^2 ^(95% CI: 30.8-49.5). In order to detect a 16 mm whole body translation, subjects with peripheral neuropathy required an acceleration of 25.3 mm/s^2 ^while subjects without peripheral neuropathy required an acceleration of 23.2 mm/s^2 ^(95% CI: 16.6-29.8).

The MANCOVA revealed no significant interaction between diabetes and peripheral neuropathy at 1 mm, 4 mm, or 16 mm displacements. There was no difference in acceleration detection thresholds between subjects with diabetes with peripheral and subjects with diabetes without peripheral neuropathy at 1 mm (148.9 mm/s^2 ^vs. 168.9 mm/s^2^), 4 mm (64.1 mm/s^2 ^vs. 66.5 mm/s^2^), or 16 mm (27.25 mm/s^2 ^vs. 39.4 mm/s^2^).

## Discussion

The results of this study provide further evidence of abnormal postural control in individuals with diabetes. Subjects with diabetes, both with and without peripheral neuropathy, required faster accelerations in order to detect fairly small (1 and 4 mm), whole body anterior translations compared to subjects without diabetes in the absence of visual information. These findings suggest that in situations with low or no light, individuals with diabetes may not be able to detect small perturbations of the surface on which they stand or that it takes a longer movement distance before they detect the onset of a slip. Both actions could place them at an increased risk for a fall, if for instance they were to slip on a small object.

A unique aspect of our experimental protocol for studying postural control is the use of the SLIP-FALLS platform to examine psychophysical aspects of balance. Postural control mechanisms have primarily been studied under two conditions -- during quiet standing or under perturbations that are large enough to require balance reactions to maintain an upright posture. This paper takes a decidedly different approach to the study of postural control than that afforded by the more traditional biomechanical methods. For all of the experiments described here, AP and ML Centers-of Pressure data, bilateral foot pressure data, head tri-axial acceleration, kinematic and lower limb EMG data were collected. The link between and among these data and detection thresholds has been and is being continually explored by our lab to determine which sense(s) best contribute(s) to threshold detection[13,14,20,26,27]. Further, traditional biomechanics are at a loss to explain concepts borrowed from the robotics literature like dither and dead-zone control that could play a key role in human postural control. These considerations are important because our psychophysical studies are carried out peri-threshold, and have perturbations whose lengths are on the order of the "noise" of normal sway. The SLIP-FALLS platform and accompanying technologies allow the examination of postural control at the edge of psychophysical detection of movement[13,14,27]. Thus, perturbations are of a length that lie within a normal sway path length[26,28], and provide a different method of assessing postural stability.

Abnormal postural control in people with diabetes is commonly attributed to the loss of somatosensory input from the lower extremities due to peripheral neuropathy. Peripheral neuropathy may affect somatosenory input (proprioceptive and tactile) and/or motor output (reaction time and strength). Using center-of-pressure measures to assess postural control during quiet standing, several studies have demonstrated that people with diabetes and peripheral neuropathy have impaired balance[1,2,6,29-31]. Simoneau et al[30], Uccioli et al[6] and DiNardo et al[29] all found that people with diabetes and peripheral neuropathy exhibited increased postural sway compared to individuals with diabetes without peripheral neuropathy and healthy controls. These researchers also reported no difference in measures of static postural control between individuals with diabetes without peripheral neuropathy and healthy controls. We reported similar findings using a composite index for measuring quiet standing postural sway based on anterior-posterior (AP) mean power, AP mean sway distance, and AP root mean square of sway distance[26].

Our current study involving very short perturbations leads to a slightly different finding, with an important distinction. It would seem that diabetes itself was the significant main effect in subjects' ability to detect small postural disturbances. It is also interesting that there was no significant difference in acceleration detection threshold between the individuals with peripheral neuropathy and those without. This difference is likely due to the conditions under which postural control and how postural control was assessed (psychophysical) between this study and others[6,26,29,30]. Other researchers[6,26,29,30] examined postural control under static conditions (quiet standing) and used biomechanical measures of postural control, while we examined psychophysical aspects of postural control under a dynamic condition (small perturbation). Detecting small postural disturbances may be a more challenging task than standing quietly. Identifying small postural disturbances at the edge of psychophysical detection requires a complex interaction of attentional processes, integration of sensory information, and neuromuscular activation.

The results of our study support a recent review by Bonnet and colleagues[10] who found that abnormal postural control in people with diabetes may be partly attributed to diabetes per se. Other authors have reported similar findings. Centomo et al[9] reported abnormal postural control in individuals with diabetes without peripheral neuropathy compared to healthy control subjects and Allet and colleagues[7] found that people with diabetes without peripheral neuropathy demonstrated abnormal gait parameters compared to healthy individuals with no difference in gait parameters between individuals with diabetes with and without peripheral neuropathy. Both of these authors concluded that diabetes per se could have a direct effect on postural control and gait and increase fall risk in people with diabetes without peripheral neuropathy. Although the methods for assessing postural control (psychophysical) are different in our study, we also examined postural control under dynamic conditions (a small perturbation) and found that diabetes itself may have an impact on postural control as well.

A possible reason for why individuals with diabetes required greater accelerations to detect whole body movements at short displacements (≤ 16 mm) is the growing evidence that diabetes can affect vestibular function[32-34]. The vestibular system is sensitive to altered blood glucose and insulin levels. Alterations in blood glucose and insulin levels in people with diabetes may impair the function of the vestibular system making it difficult for them to detect minor postural disturbances. However, our acceleration values are often below that needed for vestibular system activation[35], so the exact role of the vestibular system in detecting the short perturbations employed by this study is not known at this time. Future work will involve the direct testing of the vestibular system to explore its relative contribution.

Since our method of assessing psychophysical thresholds of balance requires attention, mild cognitive impairments secondary to diabetes could be involved[36,37]. A valid test of cognitive function in future studies is warranted. Future research could also use a dual-task paradigm (e.g., using distracters) to possibly identify an attentional component that may impact postural control in people with diabetes.

Our results indicating that diabetes itself may have an impact on the ability to detect small postural disturbances should be examined with some caveats. Even though our data met the criteria necessary for using a 2 × 2 factorial MANCOVA to detect a difference in psychophysical detection of a small whole body acceleration there was a small number of subjects with diabetes without peripheral neuropathy. There were also 14 individuals (seven DPN, five PNNoDM, and 2 NINoD) who could not identify an acceleration threshold over the course of the 30 trials in two of the three distances (1 mm, 4 mm, and 16 mm) and were not used in the analysis. There are a few possible explanations for this. A small number of these subjects appeared to be iterating towards a threshold detection that was slightly above the rail ceiling, but could not identify a threshold using the PEST procedures at or below the rail value. A few subjects appeared to have difficulty understanding the 2AFC psychophysical test procedures and did not iterate to detection threshold, even though their MMSE screening scores were within normal range and even thought they had successfully completed the training task run before each test at a given displacement.

## Conclusion

Our findings suggest diabetes itself may negatively influence the postural control system and that peripheral neuropathy may not be the sole cause of balance impairment in people with diabetes. In addition to impaired postural control under static testing conditions, we found that individuals with diabetes exhibited an impaired ability to detect short, whole body anterior translations. Clinicians should be aware that individuals with diabetes at an early stage of the disease process when they do not yet have peripheral neuropathy may have impaired balance, which may place them at risk for a fall.

## Competing interests

The authors declare that they have no competing interests.

## Authors' contributions

GDF aided in data analysis, and wrote the manuscript. CJR developed the study design, over saw data collection, aided in data analysis and drafting and revising the manuscript. SM performed data analysis and aided in drafting and revising the manuscript. CMS performed data acquisition, aided in data analysis and drafting the manuscript. AMH aided in data acquisition, subject recruitment and drafting the manuscript. All authors read and approved the final manuscript.
